# Bibliometric analysis of research trends and characteristics of drug-induced gingival overgrowth

**DOI:** 10.3389/fpubh.2022.979861

**Published:** 2022-09-06

**Authors:** Ruonan Zhang, Jie Wu, Junyi Zhu, Xiaoxiao Wang, Jiangyuan Song

**Affiliations:** ^1^Department of Stomatology, Union Hospital, Tongji Medical College, Huazhong University of Science and Technology, Wuhan, China; ^2^Hubei Province Key Laboratory of Oral and Maxillofacial Development and Regeneration, Wuhan, China; ^3^School of Stomatology, Tongji Medical College, Huazhong University of Science and Technology, Wuhan, China; ^4^Research Center for Clinical Epidemiology, Peking University Third Hospital, Beijing, China

**Keywords:** bibliometric, citation analysis, research trends, drug-induced gingival overgrowth, Scopus

## Abstract

**Objectives:**

Drug-induced gingival overgrowth (DIGO) is a frequent adverse medication reaction that is generally caused by cyclosporine, phenytoin, and nifedipine, which belong to the category of immunosuppressants, anticonvulsants, and calcium channel blockers, respectively. This bibliometric analysis aims to depict the main citation characteristics and analyze the research trends in DIGO investigations.

**Methods:**

An exhaustive search was performed in the Scopus database to create the bibliometric list of DIGO in the syntax. Furthermore, the information related to the number of citations, drugs related to DIGO, study topic and design, authorship, publication year, journal, contributing institution, country of origin, and the department was extracted.

**Results:**

In total, 399 papers on DIGO were retrieved in this study. The total number of citations and that after the removal of self-citations were 7,814 and 7,314, respectively. The mean number of citations was 19.6 in a range of 0–608. The main paper types were articles (76.94%) and reviews (19.55%). A remarkable increasing trend in the number of citations has been observed since 1994. Cyclosporine (44.89%) is the most commonly used drug that shares a close relationship with DIGO, followed by phenytoin (18.22%), nifedipine (17.93%), and amlodipine (6.81%). The review (27.82%) type constituted the most widely used design in the DIGO studies. According to the top 20 keywords, the risk factors and pathogenesis of DIGO have been prominent topics of research works for several years.

**Conclusions:**

This bibliometric analysis will facilitate the understanding of researchers and clinicians, especially those at the beginning of their careers in periodontology on DIGO, by identifying landmark research and providing an overview of this field.

## Introduction

Drug-induced gingival overgrowth (DIGO) is a systemic medication–induced side effect. The most common drugs related to DIGO are cyclosporine, phenytoin, and nifedipine, which belong to the categories of immunosuppressants, anticonvulsants, and calcium channel blockers, respectively. It is estimated that one million residents of North America are likely to be affected by DIGO (1), and this number is growing with time. It is recommended that oral health should be included as part of a care plan for patients with cardiovascular diseases receiving treatment with the suspected drugs (2), because gingival enlargement, tooth malposition (3), and long-term tooth loss are readily involved in patients with DIGO (4). DIGO management includes oral hygiene instructions, professional scaling and root planning (5), surgical intervention, and withdrawal or substitution of the causative drug (1). However, the recurrence of DIGO is relatively common in cases in which drug cessation or replacement is impossible (4). Therefore, studies in this field should be encouraged to generate novel information for developing proper preventative and therapeutic strategies.

Bibliometric analysis is a type of citation analysis that collects citation data to evaluate the scientific influence of a paper in its particular field (1). Citation data (i.e., data on references cited in footnotes or bibliographies of scholarly research publications) could explain the “impact,” “influence,” or “quality” of scholarly works. The number of citations of a paper indicates researchers' interest in using the data to conduct their studies (6). Besides, a bibliometric analysis could also help researchers identify a field's trends and hotspots. Thus, bibliometric analysis is a valuable tool for researchers and clinicians to conduct relevant research and implement clinical decisions.

However, no bibliometric analysis on DIGO has been published. Therefore, a bibliometric analysis was performed in this field to determine the top 20 articles. Furthermore, the research trends and hotspots of DIGO and characteristics of articles on DIGO, including the number of citations, study topic and design, authorship, year of publication, journal, contributing institution, country of origin, and department, were also investigated in this analysis.

## Materials and methods

The Scopus citation index was used to find the citation information about the published articles on DIGO (6). Using the search strategy [TITLE-ABS-KEY (drug-induced gingival overgrowth) OR (drug-induced gingival hyperplasia) OR (drug-induced gingival enlargement) OR (drug-induced gingival proliferation) OR (drug-induced hypertrophic gingivitis)], the Scopus database was searched from 1971 to January 6, 2021. Notably, the papers' language, study type, and design were not restricted. In total, 431 papers were retrieved from the Scopus database using the abovementioned search strategy. All the papers were arranged in descending order according to their citation counts. In cases of papers with the same number of total citations, the papers with the highest citation density were positioned higher in the ranking. Next, the titles, abstracts, or full texts of these papers were evaluated to confirm their relevance to DIGO by two independent authors (RNZ and JW). The following information was recorded: paper type, publication title, publication year, the number of papers by year, number of citations and self-citations, citation density [mean number of citations per year = total number of citations/years since the publication of the article (7)], keywords, design, area of study, author, institution, country or region of origin, name of the journal, journal impact factor (IF), quartile [2019 Journal of Citation Reports (JCR): Science Edition], level of evidence (8–10), and the involved departments. In case of disagreements, the suggestion of a third author (JYS) was required to achieve consensus. This research did not need any animal or human subjects to acquire the ethics committee's approval.

## Results

### Citation characteristics of the articles included

**Figure 1:**
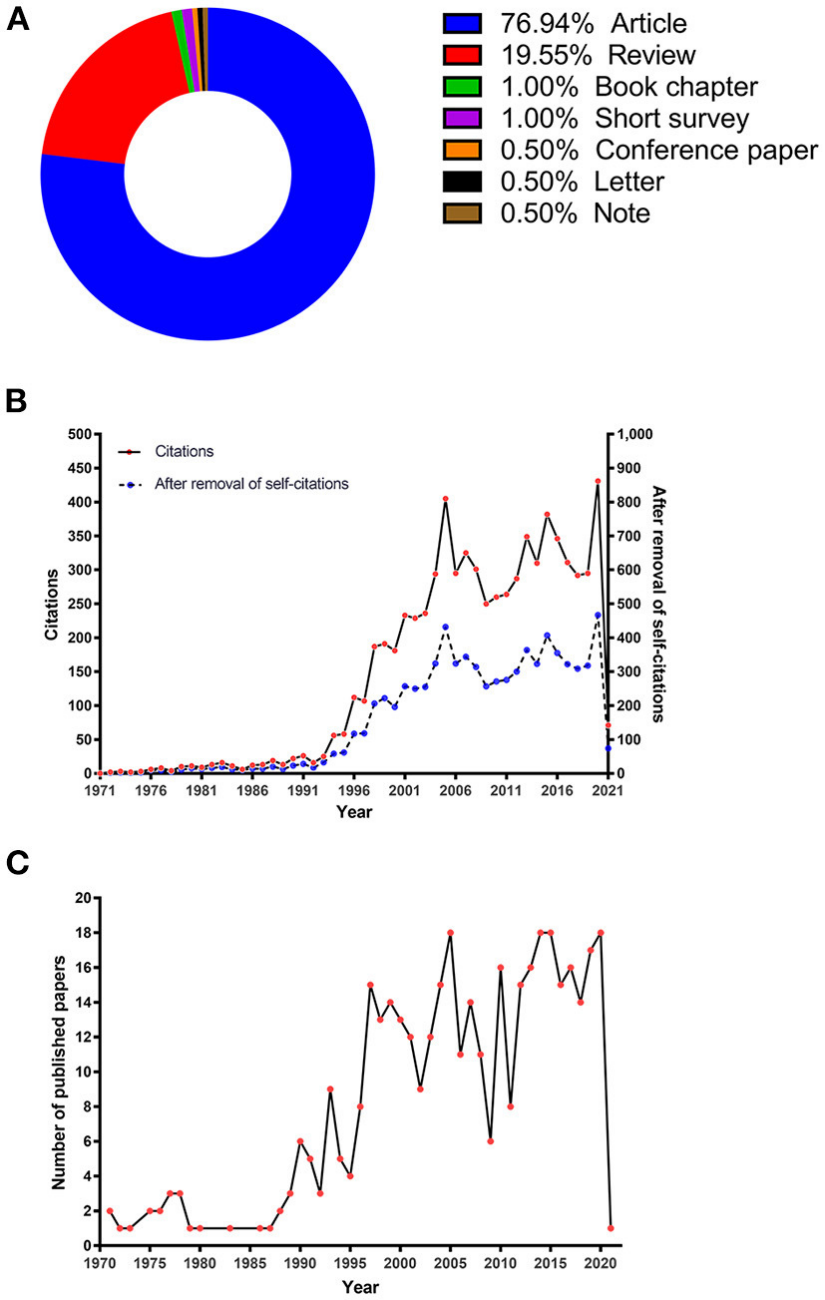
Citation characteristics of the papers on DIGO. **(A)** The frequency of paper types. **(B)** The number of citations and that after removal of self-citations of papers by year of publication. **(C)** The number of papers included by year of publication.

From 1971 (the year of the first publication) to January 6, 2021 (the time of the search), 431 papers were retrieved from the Scopus database. After eliminating the non-relevant papers, 399 research articles were included for quantitative analyses. Of the 399 articles, 76.94% were original articles, 19.55% were reviews, 3.5% were other types of articles (book chapter: 1.0%, short survey: 1.0%, conference papers: 0.5%, letters: 0.5%, and notes: 0.5%) (Figure 1A). Citation characteristics are summarized based on 333 papers with available citation data. Figure 1B shows total citations and the citations after removing self-citations each year. A remarkable increasing trend in the number of citations has been observed since 1994. Figure 1C shows the number of papers on DIGO by the year of publication. The citation trend is similar to that of the number of papers published each year.

The total number of citations and that after the removal of self-citations were 7,814 and 7,314, respectively. The number of citations in these papers ranged between 0 and 608, with a mean of 19.6 citations per paper. Table 1 presents the general information of the top 20 cited articles, including the ranking, first author, title, year and journal of publication, number of citations, citation density, and the article type. The most cited article with 608 citations was by Mayer et al., who published their research in the *Transplantation* in 1997. Their paper was a multicenter randomized trial demonstrating that DIGO occurred more frequently with cyclosporine. In addition, DIGO has attracted more and more attention since then. Their research trial also has the highest citation density (25.33) in terms of citation density. The top 2 articles were cited more than 400 times, with 13 highest-ranked articles in the list receiving more than one-third of the total citations. The top 20 cited articles were published from 1976 to 2004, including 12 reviews and 8 articles. Over half of the articles (*n* = 13) emphasized the pathogenesis of DIGO. These articles also highlighted the need to thoroughly understand the mechanism of DIGO. The common risk factors involved in the papers comprised age, gender, drug variables, periodontal variables, and genetic factors. The basic research works aimed at the mechanism of DIGO mentioned in the top 20 cited articles mainly focused on the cellular function and cell subpopulation heterogeneity of fibroblasts.

**Table 1:** Top-20 most cited papers on DIGO.

**Rank**	**First author**	**Title**	**Year of publication**	**Journal (abbreviated name)**	**Type**	**Citations**	**Citation density**
1	Mayer et al.	Multicenter randomized trial comparing tacrolimus (FK506) and cyclosporine in the prevention of renal allograft rejection: A report of the European tacrolimus multicenter renal study group	1997	Transplantation	Article	608	25.33
2	Faulds et al.	Cyclosporin: A Review of its Pharmacodynamic and Pharmacokinetic Properties, and Therapeutic Use in Immunoregulatory Disorders	1993	Drugs	Review	463	19.25
3	Seymour	The pathogenesis of drug-induced gingival overgrowth	1996	J. Clin. Periodontol.	Review	272	11.33
4	Hassell et al.	Drug-induced gingival overgrowth: Old problem, new problem	1991	Crit. Rev. Oral Biol. Med.	Review	209	8.71
5	Seymour et al.	Risk factors for drug-induced gingival overgrowth	2000	J. Clin. Periodontol.	Review	188	7.83
6	Hassell et al.	Diphenylhydantoin (dilantin) gingival hyperplasia: drug induced abnormality of connective tissue	1976	Proc. Natl. Acad. Sci. U. S. A.	Article	151	6.29
7	Herranz et al.	Clinical Side Effects of Phenobarbital, Primidone, Phenytoin, Carbamazepine, and Valproate During Monotherapy in Children	1988	Epilepsia	Article	125	5.21
8	Ilhan Uzel et al.	Connective Tissue Growth Factor in Drug-Induced Gingival Overgrowth	2001	J. Periodontol.	Article	120	5
9	Abdollahi et al.	A review of drug-induced oral reactions	2003	J. Contemp. Dental Pract.	Review	119	4.96
10	Asconapé	Some common issues in the use of antiepileptic drugs	2002	Semin. Neurol.	Review	119	4.96
11	Trackman et al.	Connective tissue metabolism and gingival overgrowth	2004	Crit. Rev. Oral Biol. Med.	Review	111	4.625
12	–	Informational paper: Drug-associated gingival enlargement	2004	J. Periodontol.	Review	104	4.63
13	Nishikawa et al.	Pathogenesis of Drug-Induced Gingival Overgrowth. A Review of Studies in the Rat Model	1996	J. Periodontol.	Review	103	4.29
14	Tipton et al.	Fibroblast heterogeneity in collagenolytic response to cyclosporine	1991	J. Cell. Biochem.	Article	93	3.88
15	Butler et al.	Drug-induced gingival hyperplasia: phenytoin, cyclosporine, and nifedipine	1997	J. Am. Dent. Assoc.	Article	93	3.88
16	Brown et al.	On the mechanism of drug-induced gingival hyperplasia	1991	J. Oral Pathol. Med.	Review	89	3.71
17	Schincaglia et al.	Cyclosporin-A increases type I procollagen production and mRNA level in human gingival fibroblasts in *vitro*	1992	J. Oral Pathol. Med.	Review	87	3.63
18	Iacopino et al.	Phenytoin and Cyclosporine A Specifically Regulate Macrophage Phenotype and Expression of Platelet-Derived Growth Factor and Interleukin-1 in *Vitro* and in Vivo: Possible Molecular Mechanism of Drug-Induced Gingival Hyperplasia	1997	J. Periodontol.	Article	85	3.54
19	Seymour et al.	Drugs and the periodontium	1988	J. Clin. Periodontol.	Review	80	3.33
20	Marshall et al.	A clinical review of drug-induced gingival overgrowths	1999	Aust. Dent. J.	Review	78	3.25

### Topic, design, and area of study of the included papers

**Figure 2:**
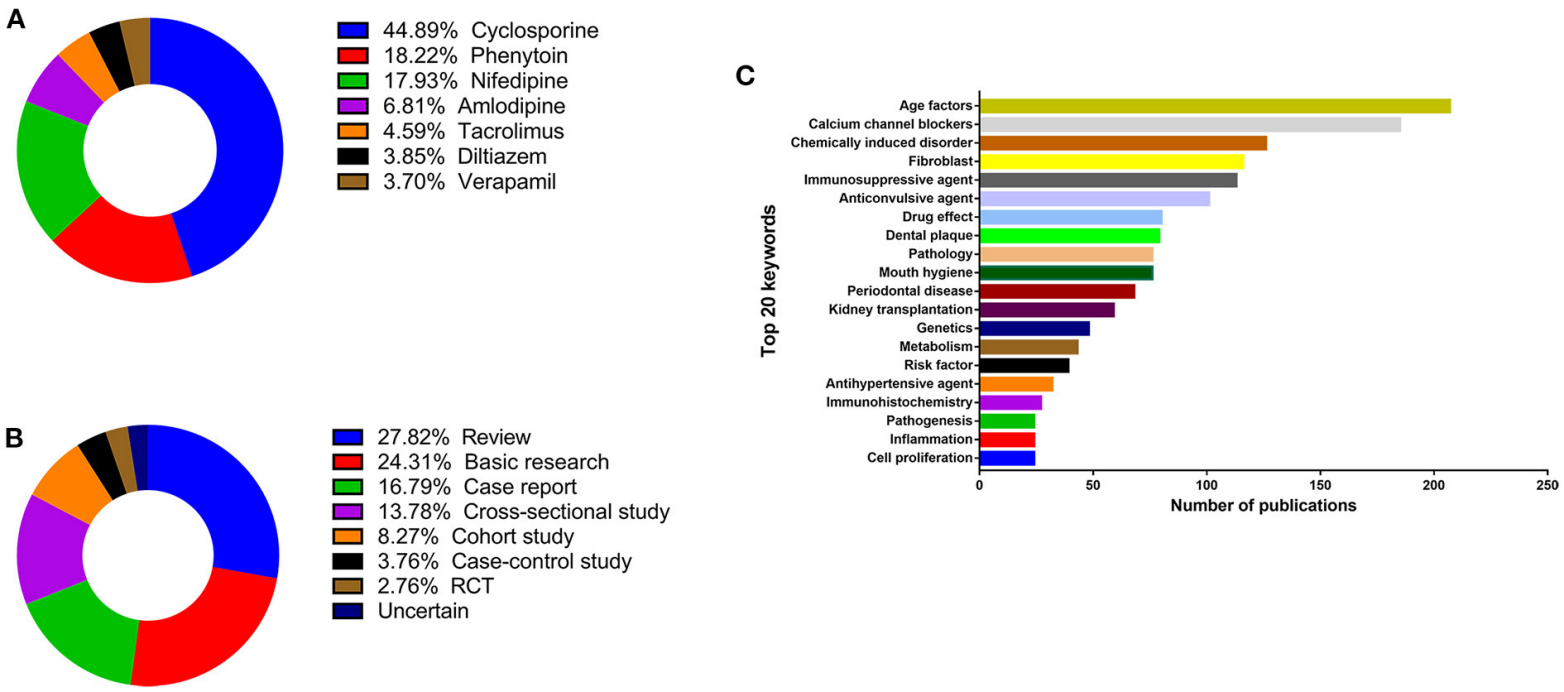
Research trend and hotspots of DIGO. **(A)** The frequency of drug related to DIGO. **(B)** The frequency of study design. **(C)** Top-20 keywords.

The frequency of keywords in each included paper was collected and analyzed to identify the research trends and hotspots of DIGO, including the study topic, design, and area of the study of essays about DIGO. For the commonly used drug, cyclosporine (44.89%) shares a close relationship with DIGO (Figure 2A), followed by phenytoin (18.22%), nifedipine (17.93%), and amlodipine (6.81%). For the study design, the review (27.82%) type constitutes the most widely used design in the DIGO study (Figure 2B), followed by basic research (24.31%), case report (16.79%), cross-sectional study (13.78%), cohort study (8.27%), case-control study (3.76%), and randomized controlled trial (RCT, 2.76%). After removing and combining some relevant keywords, age (*n* = 207), dental plaque (*n* = 79), mouth hygiene (*n* = 76), and periodontal disease (*n* = 68) are the most common keywords that may contribute to the occurrence of DIGO. According to the top 20 keywords (Figure 2C), calcium channel blockers (*n* = 185), chemically induced disorders (*n* = 126), immunosuppressive agents (*n* = 113), anticonvulsive agents (*n* = 101), and antihypertensive agents (*n* = 32) may be classified as the category of drugs that are related to gingival overgrowth. In addition, the specific research topics such as fibroblasts (*n* = 116), pathology (*n* = 76), genetics (*n* = 48), and metabolism (*n* = 43) are also presented in the list.

### Authors, institution, country or region of origin, journal of publication, and department

**Figure 3:**
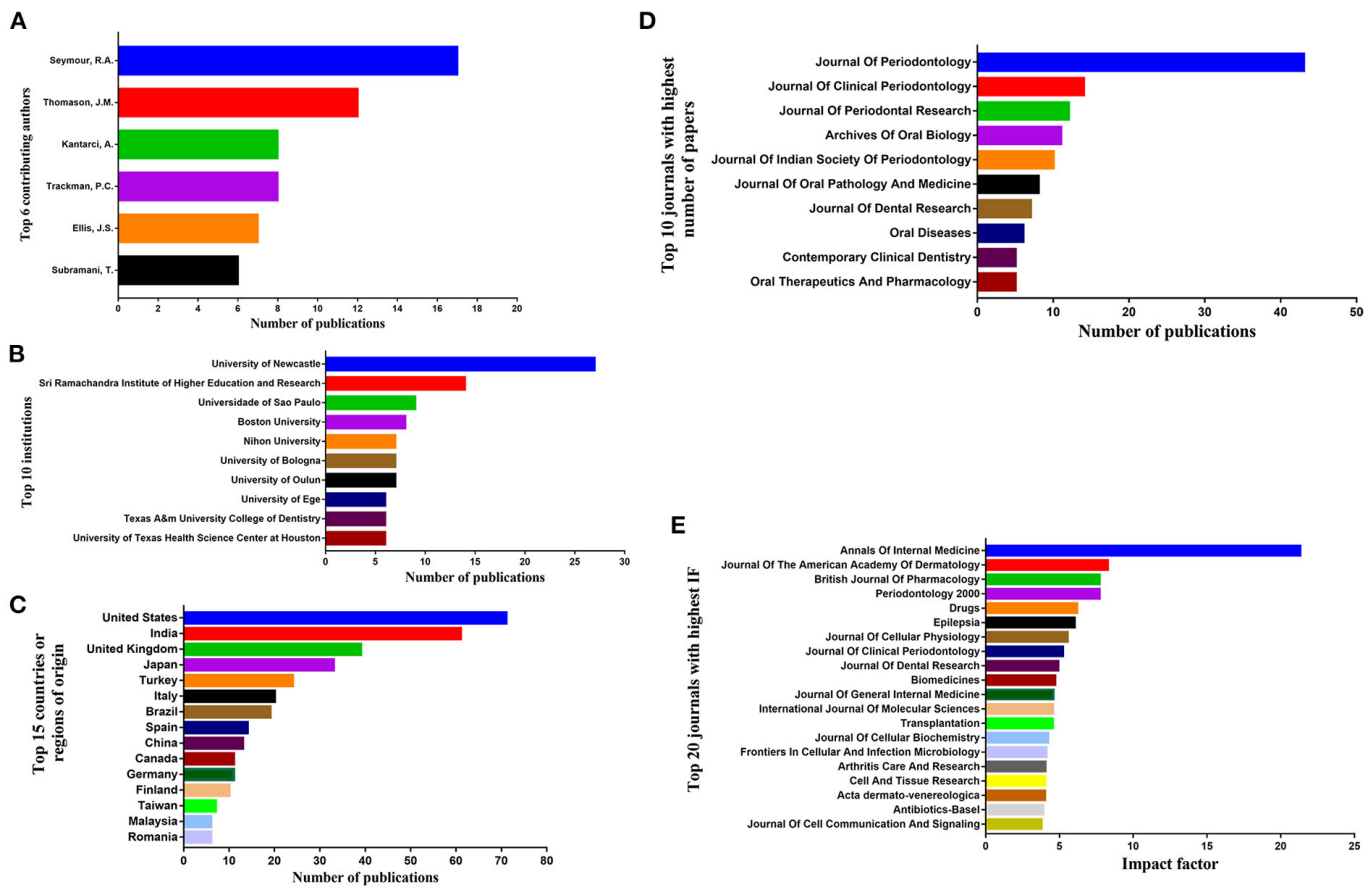
The information on the **(A)** author (rank 1–6), **(B)** institution (rank 1–15), **(C)** country or region of origin (rank 1–15), **(D)** journal of publication (rank 1–10) with the largest number and **(E)** journal with high IF (rank 1–20).

The most productive authors are “Seymour R.A.” with 17 published articles, followed by “Thomason, J.M.” with 12 published articles, who are also the only two authors that contributed to over 10 papers (Figure 3A). Most of all authors included in this review published two papers (102 authors). In terms of the first author, Seymour R.A. also tops the list with seven published articles, followed by Subramani, T. with six published articles.

There are 159 institutions, 47 of which published 3 or more papers. The University of Newcastle contributed the largest number of papers (*n* = 27), followed by the Sri Ramachandra Institute of Higher Education and Research (*n* = 14) and the University of São Paulo (*n* = 9) (Figure 3B). The contributing country with the largest number of papers is the United States (*n* = 71), followed by India (*n* = 61), the United Kingdom (*n* = 39) and Japan (*n* =33) (Figure 3C). More than half of the articles are published in the four highest contributing countries.

The papers were published in 175 journals, 83 of which are SCI journals, where nearly half of the papers (*n* = 199) were published. The *Journal of Periodontology* (*n* = 43) tops the list of journals of the number of publication, followed by the *Journal of Clinical Periodontology* (*n* = 14), the *Journal of Periodontal Research* (*n* = 12), the *Archives of Oral Biology* (*n* = 11), and the *Journal of Indian Society of Periodontology* (*n* = 10) (Figure 3D). For the 2019 journal impact factor (JIF), the *Annals of Internal Medicine* (IF = 21.317) and the *Journal of The American Academy of Dermatology* (IF = 8.277) were the two journals with the highest IF. However, when it comes to the SCI journals in stomatology, the one with the highest IF is *Periodontology* 2000 (IF = 7.718) (Figure 3E). Journal rankings in each subject category are divided into quartiles by the SCImago Journal Rank. There are four quartiles: Q1–Q4, which rank the journals from highest to lowest, educed from their impact index (7). According to 2019 JCR, 26 journals were divided into Q1, 21 into Q2, 14 into Q3, and 22 into Q4.

**Figure 4:**
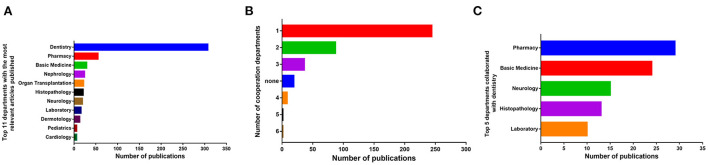
Collaborative studies of DIGO. **(A)** Top-11 departments that have published the most relevant articles. **(B)** Number of cooperation departments per article. **(C)** Top-5 departments collaborated with dentistry. none: No source of department information.

The involved departments were evaluated per article. Dentistry published the most articles (*n* = 307), followed by pharmacy (*n* = 55), basic medicine (*n* = 29), nephrology (*n* = 24), and organ transplantation department (*n* = 22) (Figure 4A). Collaborative studies were also observed as defined by the number of cooperation departments per article and the situation of collaborations with dentistry. There were six departments to cooperate at most, and 244 papers were completed by a single department (Figure 4B). The department that collaborates most with the dentistry was pharmacy (*n* = 29), followed by basic medicine (*n* = 24), nephrology (*n* = 15), histopathology (*n* = 13), and laboratory (*n* = 10) (Figure 4C).

## Discussion

This bibliometric analysis presents one of the first efforts to identify the top 20 cited articles and their main characteristics and research trend in DIGO. Seymour et al. are the most productive authors, and the research of Mayer A. D et al. is the most cited article in DIGO. The University of Newcastle is the most productive institution, and America contributes the most articles in this field. The risk factors and pathogenesis of DIGO are the two major hotspots in DIGO. These bibliometric analyses can offer insights into the research status within DIGO, identify strengths and limitations, and highlight articles that can assist researchers, trainees, and clinicians.

Only a minority of the papers (RCT: 2.76%) have a high evidence level according to the evidence pyramid made by various libraries (8–10), and there is no systematic review or meta-analysis. The majority of DIGO papers have a low evidence level (i.e., review, basic research, and case report). This phenomenon might imply a scarcity of high-level evidence in DIGO, suggesting an urgent need to conduct more RCTs. However, such studies may require multicenter collaborations, a lot of personnel, large funding, and patients' consent to receiving either an experimental or no intervention (11). Based on these high-quality RCTs, a high-quality systematic review may be available to guide clinicians, dentists, and patients on how to use the drugs to avoid or alleviate gingival overgrowth (GO) as far as possible. The advisable RCTs should include information such as the specific medication, the intervention (either withdraw the medication and/or professional dental cleaning and scaling), the case selection, the outcome and so forth, to establish a standard treatment protocol for DIGO and create an algorithm to identify the patients with suspected DIGO before taking the causal drugs.

The research trend and hotspots of DIGO were ascertained by analyzing the keywords and the contents of the included papers. Unsurprisingly, the top three frequency drugs related to DIGO were cyclosporine (44.89%), phenytoin (18.22%), and nifedipine (17.93%). However, it is noteworthy that amlodipine (6.81%), tacrolimus (4.59%), diltiazem (3.85%), and verapamil (3.70%) are also related to DIGO. Hence, amlodipine might not always be an appropriate substitute for nifedipine in patients with DIGO. The risk factors and pathogenesis are the two major hotspots of DIGO (Figure 2C). Risk factors involved with DIGO are age factors, medication (calcium channel blockers, immunosuppressive agents, anticonvulsant agents, etc.), periodontal variables (dental plaque, mouth hygiene, periodontal disease, inflammation), and genetics (12); these are also top 20 frequency keywords in this review. Because risk factors are associated with the prevalence and severity of DIGO, clinicians and dentists should be aware of these factors to help patients avoid these side effects and develop optimal management strategies. The pathogenesis of DIGO is complicated, obscure, and has been a hot research topic for several years. Fibroblast plays an essential role in the pathogenesis of DIGO. Because not all patients receiving these systemic medications develop GO, the researchers have hypothesized that the suspected individuals have fibroblasts with an abnormal susceptibility to the drug (1). Besides, differential proportions of fibroblast subsets in each individual exhibit susceptibility or resistance to pharmacologically induced gingival enlargement (13, 14). This viewpoint further demonstrated that cyclosporine A (CsA) could react with a phenotypically distinct subpopulation of gingival fibroblasts to enhance the protein synthesis (15, 16). In addition to the role of fibroblast, other factors (i.e., inflammatory cytokines, matrix metalloproteinase) may also contribute to the pathogenesis of DIGO (17).

The United States (US) has contributed the most articles on DIGO. The top-ranking position of the US is not unique to THE DIGO research and is visible in many other fields (18–22). This result indicates that countries with greater economic backgrounds tend to conduct more biomedical research, perhaps because of better medical and scientific resources and funding (23). The number of papers on DIGO published in the past two decades accounted for nearly two-thirds of the total papers. As the high prevalence and severity of DIGO become increasingly apparent, more attention is focused on it, resulting in the publication of more articles.

The fact that 175 journals are not all in the dental field reflects the increasingly multidisciplinary research works of DIGO and the need to maximize the impact of the relevant research. Unexpectedly, most of the top-cited articles were published in the *Journal of Periodontology* (IF = 3.742) and *Journal of Clinical Periodontology* (IF = 5.241), both of which are considered the leading journals related to all aspects of research and clinical practice in periodontology. This outcome indicates that the investigators often select the two journals most frequently to publish and obtain DIGO information. In general, researchers tend to publish their articles and collect related information on the reference of IF and quartile. Most of the highly cited articles and high-IF journals come from North America or Europe. The trend of bibliometric analysis is in accordance with Bradford's law (24).

The analysis of the involvement of the departments in DIGO revealed that the majority of the cooperation is focused on dentistry alone. Pharmacy and basic medicine are the two prominent departments collaborating with dentistry. As it is known, the most common drugs related to DIGO are cyclosporine, phenytoin, and nifedipine, which precisely correspond with 3 (organ transplantation, neurology, and cardiology) of the top 11 departments that have published the most relevant articles (Figure 4C). Therefore, the condition of collaboration with dentistry fits in the trend of research fields. There is still a lot of space for multidisciplinary team cooperation in the DIGO research on how to avoid or relieve the prevalence or severity of GO, such as by determining the timing between medication and periodontal treatment and the time interval of periodontal treatment.

In this review, several limitations are present in the bibliometric analysis of DIGO. First, only the Scopus database was used to determine the number of citations of the articles. It has been suggested that searching in more than one database may offer a more comprehensive view of the citations because the number of citations of the same article may differ across the databases (i.e., Google Scholar and Web of Science) (25). However, the Scopus database could automatically exclude self-citing and provide about 20% more coverage than the Web of Science, whereas Google Scholar provides results having inconsistent accuracy (26). Second, an inherent limitation of the citation analysis is the lack of correction for self-citations or the potential bias because the authors cite papers from journals in which they hope to publish their works (27). Third, citation analyzers tend to cite previous highly cited papers with a time accumulation, causing potential bias. Thus, the citation density index was used to control this potential bias, which was strongly correlated with the absolute number of citations. Fourth, the citation count does not directly reflect a paper's quality. In this study, the majority of papers provide the lowest level of evidence (i.e., review, case report, and so on).

This is the first article to report the bibliometric characteristics and research trends in terms of evidence-based dentistry regarding DIGO. Despite its limitations, this bibliometric analysis will facilitate the understanding of researchers and clinicians, especially those at the beginning of their careers in periodontology on DIGO, by identifying landmark research and providing an overview of this field. More high levels of scientific evidence on DIGO, such as systematic reviews and randomized controlled trials, are encouraged to serve as a valuable tool in reducing DIGO's incidence and severity.

## Author contributions

JS contributed to conception and design of the study. RZ and JW carried out the literature search, performed data analysis, contributed to the interpretation of data, and prepared the main part of the manuscript. JS, XW, and JZ contributed to revising and supervising of the manuscript. All authors approved the final version of the manuscript and accept accountabilities for all aspects of the work, declaring that questions related to the accuracy or integrity of all parts of the work have been appropriately investigated and reported.

## Funding

This work was supported by the National Natural Science Foundation of China (82101264).

## Conflict of interest

The authors declare that the research was conducted in the absence of any commercial or financial relationships that could be construed as a potential conflict of interest.

## Publisher's note

All claims expressed in this article are solely those of the authors and do not necessarily represent those of their affiliated organizations, or those of the publisher, the editors and the reviewers. Any product that may be evaluated in this article, or claim that may be made by its manufacturer, is not guaranteed or endorsed by the publisher.
